# Supplementary material for: Unveiling the Bioactive Potential of the Invasive Jellyfish Phyllorhiza punctata Through Integrative Transcriptomic and Proteomic Analyses
Source: Biomolecules. 2025 Aug 4;15(8):1121. doi: 10.3390/biom15081121 (PMC12383608; doi:10.3390/biom15081121)
Supplement: Supplementary file 1 [file biomolecules-15-01121-s001.zip › Suplementary Material/FileS3/PEPFOLD4/XP_065060000_6254/clusters.html]

 PEP-FOLD clusters


Download archive of all models  here   

|  |  |  |
| --- | --- | --- |
| # | file | sOPEP |
| # cluster 1 - Size: 14 |  |  |
|  | XP\_065060000\_6254-model1 | -24.2297 |
|  | XP\_065060000\_6254-model1.1 | -23.9894 |
|  | XP\_065060000\_6254-model1.2 | -23.8982 |
|  | XP\_065060000\_6254-model1.3 | -23.8021 |
|  | XP\_065060000\_6254-model1.4 | -23.7848 |
|  | XP\_065060000\_6254-model1.5 | -23.5254 |
|  | XP\_065060000\_6254-model1.6 | -23.5128 |
|  | XP\_065060000\_6254-model1.7 | -23.4339 |
|  | XP\_065060000\_6254-model1.8 | -23.4155 |
|  | XP\_065060000\_6254-model1.9 | -23.3579 |
|  | XP\_065060000\_6254-model1.10 | -23.2098 |
|  | XP\_065060000\_6254-model1.11 | -23.0657 |
|  | XP\_065060000\_6254-model1.12 | -22.6422 |
|  | XP\_065060000\_6254-model1.13 | -22.1131 |
| # cluster 2 - Size: 2 |  |  |
|  | XP\_065060000\_6254-model2 | -24.016 |
|  | XP\_065060000\_6254-model2.1 | -22.8967 |
| # cluster 3 - Size: 6 |  |  |
|  | XP\_065060000\_6254-model3 | -23.8648 |
|  | XP\_065060000\_6254-model3.1 | -23.7081 |
|  | XP\_065060000\_6254-model3.2 | -23.6434 |
|  | XP\_065060000\_6254-model3.3 | -23.6059 |
|  | XP\_065060000\_6254-model3.4 | -23.3815 |
|  | XP\_065060000\_6254-model3.5 | -23.0554 |
| # cluster 4 - Size: 6 |  |  |
|  | XP\_065060000\_6254-model4 | -23.5573 |
|  | XP\_065060000\_6254-model4.1 | -23.4881 |
|  | XP\_065060000\_6254-model4.2 | -23.362 |
|  | XP\_065060000\_6254-model4.3 | -23.3279 |
|  | XP\_065060000\_6254-model4.4 | -22.4212 |
|  | XP\_065060000\_6254-model4.5 | -21.4654 |
| # cluster 5 - Size: 16 |  |  |
|  | XP\_065060000\_6254-model5 | -22.7968 |
|  | XP\_065060000\_6254-model5.1 | -22.5456 |
|  | XP\_065060000\_6254-model5.2 | -22.4717 |
|  | XP\_065060000\_6254-model5.3 | -22.2973 |
|  | XP\_065060000\_6254-model5.4 | -22.023 |
|  | XP\_065060000\_6254-model5.5 | -21.8534 |
|  | XP\_065060000\_6254-model5.6 | -21.6574 |
|  | XP\_065060000\_6254-model5.7 | -21.564 |
|  | XP\_065060000\_6254-model5.8 | -21.5198 |
|  | XP\_065060000\_6254-model5.9 | -21.4872 |
|  | XP\_065060000\_6254-model5.10 | -21.4237 |
|  | XP\_065060000\_6254-model5.11 | -21.3156 |
|  | XP\_065060000\_6254-model5.12 | -21.2541 |
|  | XP\_065060000\_6254-model5.13 | -21.2249 |
|  | XP\_065060000\_6254-model5.14 | -20.7035 |
|  | XP\_065060000\_6254-model5.15 | -20.6959 |
| # cluster 6 - Size: 9 |  |  |
|  | XP\_065060000\_6254-model6 | -22.6215 |
|  | XP\_065060000\_6254-model6.1 | -22.5726 |
|  | XP\_065060000\_6254-model6.2 | -21.9199 |
|  | XP\_065060000\_6254-model6.3 | -21.7122 |
|  | XP\_065060000\_6254-model6.4 | -21.6859 |
|  | XP\_065060000\_6254-model6.5 | -21.6099 |
|  | XP\_065060000\_6254-model6.6 | -21.49 |
|  | XP\_065060000\_6254-model6.7 | -21.1788 |
|  | XP\_065060000\_6254-model6.8 | -20.539 |
| # cluster 7 - Size: 9 |  |  |
|  | XP\_065060000\_6254-model7 | -22.3963 |
|  | XP\_065060000\_6254-model7.1 | -22.1006 |
|  | XP\_065060000\_6254-model7.2 | -22.0447 |
|  | XP\_065060000\_6254-model7.3 | -21.8903 |
|  | XP\_065060000\_6254-model7.4 | -21.5501 |
|  | XP\_065060000\_6254-model7.5 | -20.9539 |
|  | XP\_065060000\_6254-model7.6 | -20.843 |
|  | XP\_065060000\_6254-model7.7 | -20.6922 |
|  | XP\_065060000\_6254-model7.8 | -20.6206 |
| # cluster 8 - Size: 2 |  |  |
|  | XP\_065060000\_6254-model8 | -22.3129 |
|  | XP\_065060000\_6254-model8.1 | -21.0693 |
| # cluster 9 - Size: 2 |  |  |
|  | XP\_065060000\_6254-model9 | -22.2458 |
|  | XP\_065060000\_6254-model9.1 | -20.6834 |
| # cluster 10 - Size: 8 |  |  |
|  | XP\_065060000\_6254-model10 | -22.2235 |
|  | XP\_065060000\_6254-model10.1 | -22.1731 |
|  | XP\_065060000\_6254-model10.2 | -21.8573 |
|  | XP\_065060000\_6254-model10.3 | -21.4279 |
|  | XP\_065060000\_6254-model10.4 | -21.3276 |
|  | XP\_065060000\_6254-model10.5 | -21.2753 |
|  | XP\_065060000\_6254-model10.6 | -21.161 |
|  | XP\_065060000\_6254-model10.7 | -20.8751 |
| # cluster 11 - Size: 3 |  |  |
|  | XP\_065060000\_6254-model11 | -22.0825 |
|  | XP\_065060000\_6254-model11.1 | -21.6297 |
|  | XP\_065060000\_6254-model11.2 | -20.8173 |
| # cluster 12 - Size: 5 |  |  |
|  | XP\_065060000\_6254-model12 | -21.7817 |
|  | XP\_065060000\_6254-model12.1 | -21.4843 |
|  | XP\_065060000\_6254-model12.2 | -21.1196 |
|  | XP\_065060000\_6254-model12.3 | -20.8708 |
|  | XP\_065060000\_6254-model12.4 | -20.5136 |
| # cluster 13 - Size: 3 |  |  |
|  | XP\_065060000\_6254-model13 | -21.7035 |
|  | XP\_065060000\_6254-model13.1 | -21.4847 |
|  | XP\_065060000\_6254-model13.2 | -20.4641 |
| # cluster 14 - Size: 1 |  |  |
|  | XP\_065060000\_6254-model14 | -21.6189 |
| # cluster 15 - Size: 5 |  |  |
|  | XP\_065060000\_6254-model15 | -21.5441 |
|  | XP\_065060000\_6254-model15.1 | -21.2826 |
|  | XP\_065060000\_6254-model15.2 | -20.718 |
|  | XP\_065060000\_6254-model15.3 | -20.6081 |
|  | XP\_065060000\_6254-model15.4 | -20.4396 |
| # cluster 16 - Size: 2 |  |  |
|  | XP\_065060000\_6254-model16 | -21.4019 |
|  | XP\_065060000\_6254-model16.1 | -19.8448 |
| # cluster 17 - Size: 1 |  |  |
|  | XP\_065060000\_6254-model17 | -21.3921 |
| # cluster 18 - Size: 2 |  |  |
|  | XP\_065060000\_6254-model18 | -21.3492 |
|  | XP\_065060000\_6254-model18.1 | -20.885 |
| # cluster 19 - Size: 1 |  |  |
|  | XP\_065060000\_6254-model19 | -21.3069 |
| # cluster 20 - Size: 1 |  |  |
|  | XP\_065060000\_6254-model20 | -21.268 |
| # cluster 21 - Size: 2 |  |  |
|  | XP\_065060000\_6254-model21 | -20.6566 |
|  | XP\_065060000\_6254-model21.1 | -20.3522 |
|  |  |  |
